# Supplementary figures and images for: Genomic prediction using low-coverage portable Nanopore sequencing
Source: PLoS One. 2021 Dec 15;16(12):e0261274. doi: 10.1371/journal.pone.0261274 (PMC8673642; doi:10.1371/journal.pone.0261274)

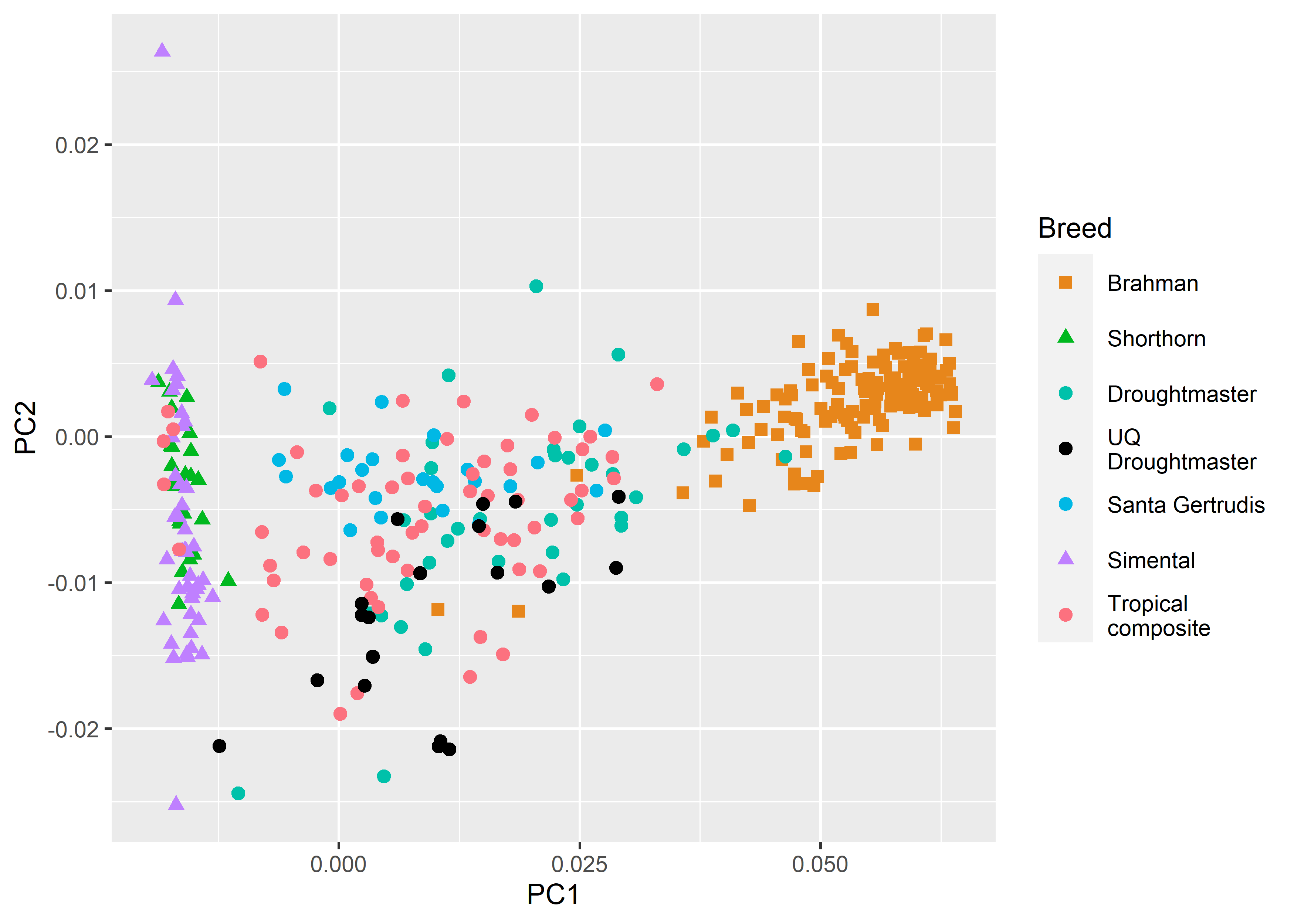

Supplement: S1 Fig — (TIF) [file pone.0261274.s001.tif]
